# Supplementary material for: Vitamin D Modified DSS-Induced Colitis in Mice via STING Signaling Pathway
Source: Biology (Basel). 2025 Jun 18;14(6):715. doi: 10.3390/biology14060715 (PMC12190092; doi:10.3390/biology14060715)
Supplement: Supplementary file 1 [file biology-14-00715-s001.zip › Supplementary Materials S1: 16s-report of gut microbiota/Groups/diff_analysis/heatmap/Genuslogdiffheatmap.pdf]

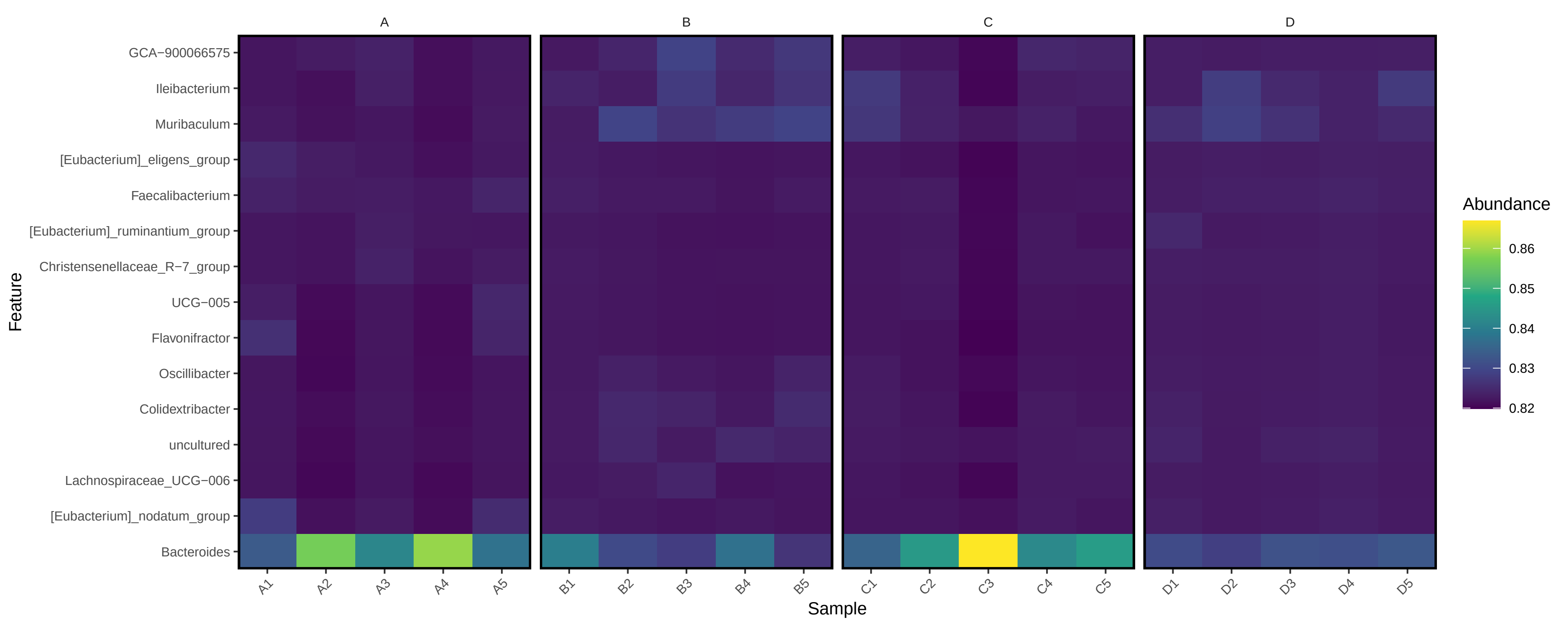

A
B
C
D
Feature
GCA−900066575
Ileibacterium
Muribaculum
[Eubacterium]_eligens_group
Faecalibacterium
[Eubacterium]_ruminantium_group
Christensenellaceae_R−7_group
UCG−005
Flavonifractor
Oscillibacter
Colidextribacter
uncultured
Lachnospiraceae_UCG−006
[Eubacterium]_nodatum_group
Bacteroides
A1
A2
A3
A4
A5
B1
B2
B3
B4
B5
C1
C2
C3
C4
C5
D1
D2
D3
D4
D5
Sample
Abundance
0.86
0.85
0.84
0.83
0.82
